# Complete Genome Sequence of a Rodent Torque Teno Virus in Hainan Island, China

**DOI:** 10.1128/MRA.01074-18

**Published:** 2018-11-15

**Authors:** Yue Wu, Shanshan Wang, Yunchun Chen, Lihua Li, Wenqi Wang, Huanhuan Zhou, You Zhang, Lei Zhang, Xiuji Cui, Gang Lv, Feifei Yin

**Affiliations:** a Hainan Medical University–The University of Hong Kong Joint Laboratory of Tropical Infectious Diseases, Hainan Medical University, Haikou, Hainan, China; b Key Laboratory of Translation Medicine Tropical Diseases, Hainan Medical University, Haikou, Hainan, China; c Department of Pathogen Biology, Hainan Medical University, Haikou, Hainan, China; d Haikou Branch, Yueyang Hospital of Integrated Traditional Chinese and Western Medicine, Shanghai University of Traditional Chinese Medicine, Shanghai, China; e Core Facility and Technical Support of Wuhan Institute of Virology, Chinese Academy of Sciences, Wuhan, Hubei, China; Iowa State University

## Abstract

Torque teno virus (TTV) has been reported in a wide range of mammals. In this study, we sequenced and analyzed the complete genome of a genetic variant of a rodent TTV, RoTTV3-HMU1 (Hainan Medical University 1). The virus was found in a rat (Rattus norvegicus) in a residential area of Hainan Island, China.

## Abstract (precis)

Torque teno virus (TTV) has been reported in a wide range of mammals. In this study, we sequenced and analyzed the complete genome of a genetic variant of a rodent TTV, RoTTV3-HMU1 (Hainan Medical University 1).

## ANNOUNCEMENT

Torque teno virus (TTV) is a nonenveloped single-stranded DNA virus (1). TTV is found widely distributed throughout the world and infects an extensive range of mammals (1–7). The possible role of TTV in disease has not been fully elucidated. Rodent TTV (RoTTV) was initially identified in the United Kingdom in populations of wild rodents in 2014, and the genotypes are RoTTV1 and RoTTV2 (8). The same research team further detected RoTTV2 in commonly used laboratory rats (9). Recently, a novel genotype of RoTTV, RoTTV3, was found in murine rodents and house shrews in 6 regions of 4 provinces in China (10).

In this study, a genetic variant of RoTTV3, RoTTV3-HMU1 (Hainan Medical University 1), was identified in a rat (Rattus norvegicus) captured in a residential area of Haikou City, Hainan Province, southern China. Viral DNA was extracted from the liver tissue with the QIAamp MinElute virus spin kit (Qiagen), and sequence-independent amplification of viral nucleic acids was performed as described previously (11). The amplicons in the 250 to 500-bp range were purified with a gel extraction kit (Tiangen). Five hundred nanograms of DNA were fragmented with Covaris S2 shearing and subjected to high-throughput paired-end 2 × 100-bp sequencing on an Illumina HiSeq 2000 instrument. After cleaning with Trimmomatic using standard parameters, reads were *de novo* assembled with Trinity version 2.0.6 (12, 13, 14). The contigs were compared with the NCBI nucleotide database, and 5 contigs (*N*_50_ = 361) were matched to RoTTV with a coverage of 0.68. Primers were designed to cover the genome by PCR amplification and Sanger sequencing. The genome was annotated with the NCBI ORFfinder and FGENESV0 (15, 16). Phylogenetic analysis was implemented with the neighbor-joining method in the MEGA6 software package (17).

The complete genome of RoTTV3-HMU1 is 2,570 nucleotides (nt) long with a G+C content of 46.93%. The genomic organization of RoTTV3-HMU1 is consistent with that of other RoTTVs, and the genome size and length of the open reading frames (ORFs) showed close similarity to those of RoTTV3. RoTTV3-HMU1 encoded 3 unidirectional overlapping ORFs. ORF1, ORF2, and ORF3 encoded proteins of 574, 79, and 98 amino acids (aa), respectively. The genome of the RoTTV3-HMU1 virus was most closely related to RN_2_Se15 (GenBank accession no. KM668486), with an identity of 95% at nucleotide level. They were also closely related at their ORF encoded proteins (ORF1, 97%; ORF2, 96%; ORF3, 89%). Phylogenetic analysis based on both ORF1 and the total genome sequence placed RoTTV3-HMU1 in the clad RoTTV3 of the RoTTV.

Hainan Island is isolated from mainland China by sea, but the same RoTTV genotype was identified on both the island and the mainland. The detection of RoTTV3-HMU1 may contribute to a better understanding of the origin and evolution of RoTTV.

### Data availability.

The genomic sequence of RoTTV3-HMU1 has been deposited in GenBank under accession no. MF688246. This whole**-**genome shotgun project and the assembly reads have been deposited in GenBank under the accession no. SRP158097 and SRR7700917, respectively.
